# Evaluation of androgen-induced effects on the uptake of [^18^F]FDG, [^11^C]choline and [^11^C]acetate in an androgen-sensitive and androgen-independent prostate cancer xenograft model

**DOI:** 10.1186/2191-219X-3-31

**Published:** 2013-04-24

**Authors:** Kimy M Emonds, Johannes V Swinnen, Evelyne Lerut, Michel Koole, Luc Mortelmans, Felix M Mottaghy

**Affiliations:** 1 Department of Nuclear Medicine, University Hospitals Leuven, Leuven 3000, Belgium; 2 Laboratory of Lipid Metabolism and Cancer, Department of Oncology, KU Leuven, Leuven 3000, Belgium; 3 Department of Morphology and Molecular Pathology, University Hospitals Leuven, Leuven 3000, Belgium; 4 Department of Nuclear Medicine, University Hospital of Aachen, Aachen 52057, Germany; 5 Department of Nuclear Medicine, Maastricht University Medical Center, Maastricht, 6202, the Netherlands

**Keywords:** Prostate cancer, Androgen deprivation, PET, [^18^F]FDG, [^11^C]choline, [^11^C]acetate

## Abstract

**Background:**

Androgen deprivation (AD) is generally used as a first-line palliative treatment in prostate cancer (PCa) patients with rising prostate-specific antigen (PSA) after primary therapy. To acquire an accurate detection of tumour viability following AD with positron emission tomography (PET), an androgen-independent uptake of tracers would be advantageous. Several metabolic PET tracers are employed for detecting recurrent PCa. We evaluated the effect of AD on the uptake of 2-deoxy-2-[^18^F]fluoro-d-glucose ([^18^F]FDG), [^11^C]choline and [^11^C]acetate *in vivo*.

**Methods:**

An [^18^F]FDG, [^11^C]choline and [^11^C]acetate baseline micro(μ)PET/μ computed tomography (CT) scan was subsequently performed in xenografts of androgen-sensitive (LAPC-4) and androgen-independent (22Rv1) tumours in nude mice. An untreated control group was compared to a surgical castration group, i.e. androgen-deprived group. μPET/μCT imaging with the above-mentioned tracers was repeated 5 days after the start of treatment. The percentage change of SUV_max_ and SUV_meanTH_ in the tumours was calculated.

**Results:**

AD did not significantly affect the uptake of [^18^F]FDG and [^11^C]choline in LAPC-4 tumours as compared with the uptake of both tracers in untreated tumours. In control 22Rv1 tumours, [^11^C]choline and [^18^F]FDG uptake increased over time. However, compared with the uptake in control tumours, AD significantly decreased the uptake of [^11^C]choline and tended to decrease [^18^F]FDG uptake. [^11^C]acetate uptake remained unaffected by AD in both PCa xenograft models.

**Conclusions:**

[^18^F]FDG and especially [^11^C]choline PET, which is currently used for the detection of recurrent PCa, could miss or underestimate the presence of local recurrent PCa following AD therapy. [^11^C]acetate uptake occurs independently of androgens and thus may be more favourable for detecting tumour viability during or following AD.

## Background

Prostate cancer (PCa) is the second most common cancer affecting approximately 17% of the male population and is worldwide becoming a considerable health issue with an increasing incidence during the last decade [1]. Primary therapy including prostatectomy or radiotherapy intends to cure this disease. Nevertheless, local and/or distal recurrences arise within 10 years in 20% to 40% of all patients. Because PCa development and proliferation is initially driven by androgens, androgen deprivation (AD) is the first-line therapy for recurrent PCa. However, over time, biochemical failure occurs and castration-resistant PCa (CRPC) develops which requires a change of treatment. Therefore, an accurate detection of tumour viability of recurrent PCa following AD is warranted.

Using conventional imaging techniques such as ultrasound, computed tomography (CT) or magnetic resonance imaging, an accurate definition of a treatment response in relapsing patients is not always evident. Therefore, molecular imaging using positron emission tomography (PET) could be advantageous in this setting since biological changes usually precede morphological alterations and an early tumour-specific response could be defined. At present, 2-deoxy-2-[^18^F]fluoro-d-glucose ([^18^F]FDG), which visualises the increased glucose demand of tumours, is generally used in oncologic PET imaging. However, [^18^F]FDG PET is less valuable in PCa due to the slow growth rate of this tumour entity. The renal excretion of the tracer and an equally increased tracer uptake in benign prostatic hyperplasia or prostatitis further hampers the detection of the primary tumour [2-4]. Some reports describe the detection of metastases by means of [^18^F]FDG PET [5]. However, to better visualise local PCa recurrences, radiolabelled choline and [^11^C]acetate have been evaluated. Choline PET imaging reflects the membrane duplication of proliferating cells, whereas [^11^C]acetate PET visualises the enhanced lipid synthesis [6] in cancer cells. In a comparative study, both tracers performed similarly [7]. Also, both tracers demonstrate a more favourable excretion pattern compared to that of [^18^F]FDG [8,9]. A correlation of choline uptake with cellular proliferation rate was not found [10]. Nevertheless, radiolabelled choline is currently applied in most centres for the detection of PCa recurrences and is being investigated for assessing therapeutic response.

The development, proliferation and maintenance of normal and malignant prostate cells are controlled by androgens, which activate androgen receptor (AR) signalling. To this end, patients relapsing after primary therapy are generally treated with AD in order to inhibit PCa proliferation and to induce apoptosis of tumour cells. Although AR signalling controls the expression of many target genes, it is not fully elucidated so far whether the uptake of metabolic PET tracers such as [^18^F]FDG, [^11^C]choline and [^11^C]acetate is substantially hampered by interfering with this molecular signalling pathway during AD. In the clinical setting, anti-hormonal therapy has been shown to reduce the [^18^F]FDG and [^11^C]choline PET signal in PCa [11-13]. Besides therapeutic effects such as suppression of tumour growth and decreased tumour viability, this observation could in part result from a therapy-induced inhibition of cellular uptake mechanisms. Also, AR signalling is ongoing in CRPC and seems to be of importance for disease maintenance. Therefore, androgen may influence gene expression patterns while tumour proliferation remains independent of AD treatment.

Thus, following the start of AD treatment at advanced stages of PCa, metabolic imaging may be affected, which could result in a disturbed detection of early treatment response and a false negative PET scan. For this purpose, we evaluated the short-term effect of AD on the uptake of [^18^F]FDG, [^11^C]choline and [^11^C]acetate in a hormone-naive and a CRPC animal model.

## Methods

### Cell lines

The human-derived PCa cell lines LAPC-4 (androgen-sensitive) [14] and 22Rv1 (androgen-independent yet androgen-responsive) [15-17] were cultured at 37°C in a humidified incubator with 5% CO_2_/95% air atmosphere. The wild-type AR-expressing LAPC-4 cell line was kindly provided by Prof. Karen Knudson (the Kimmel Cancer Center, Thomas Jefferson University, Philadelphia, PA, USA). LAPC-4 cells were grown in Iscove’s modified Dulbecco’s medium (Thermo Fisher Scientific, Waltham, MA, USA) supplemented with 10% foetal bovine serum (FBS) (Invitrogen, Life Technologies, Carlsbad, CA, USA), 2 mM l-glutamine (Invitrogen), and 100 μg/ml streptomycin and 100 U/ml penicillin (Invitrogen). The 22Rv1 cell line is a preclinical model mimicking the progression towards CRPC. The AR population of 22Rv1 is heterogeneous consisting of a constitutive active truncated AR (ARΔLBD), and a mutated - substitution of tyrosine for histidine (H875Y) in the LBD and/or an in-frame exon 3 duplication (E3DM) in the DNA-binding domain - AR. The 22Rv1 cell line (American Type Culture Collection, Manassas, VA, USA) was cultured in RPMI 1640 medium (Invitrogen) supplemented with 10% FBS and 3 mM l-glutamine.

### Animal model

Seven-week-old male athymic *nu/nu* mice were obtained from Janvier (Le Genest Saint Isle, France). The animals were housed in individually ventilated cages and kept under a 12 h light–dark cycle. Animal experiments were performed according to the strict guidelines for care and handling of laboratory animals established by the ethical committee of the KU Leuven.

PCa xenografted tumours were grown subcutaneously in both shoulders of the animal. LAPC-4 xenografted mice were obtained approximately 6 weeks after the injection of 5 × 10^6^ cells with matrigel (1:1) (BD Biosciences, San Jose, CA, USA) in a final volume of 100 μl. 22Rv1 cells were concentrated to 2 × 10^6^ cells per 100 μl, giving tumours after about 1 month. Follow-up of developing tumours was accomplished by calliper measurement, and tumour volumes (mm^3^) were calculated with the equation *l* × *w* × *h* × (*π*/6) (length (*l*), width (*w*) and height (*h*) represent the three orthogonal dimensions of the tumour in millimetres). A tumour size of at least 150 mm^3^ was requested for inclusion into the micro(μ)PET imaging study.

### Study design

**Figure 1:**
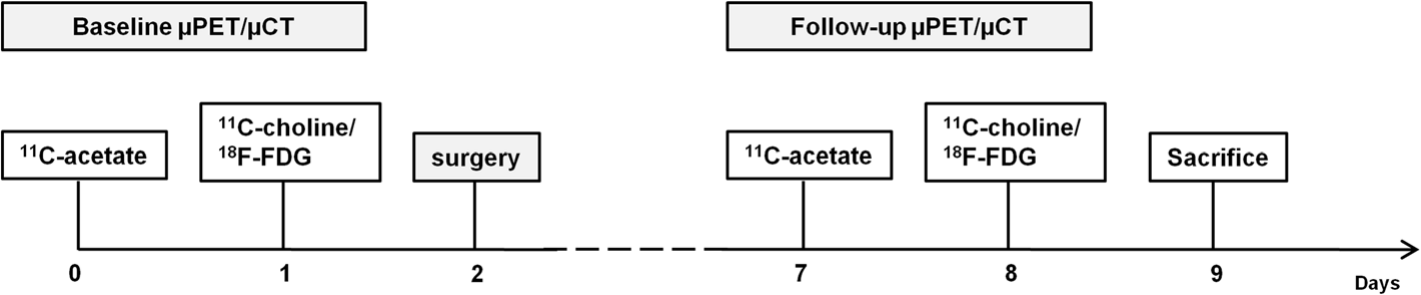
Schematic illustration of the study design.

For all mice, an [^18^F]FDG, [^11^C]choline and [^11^C]acetate baseline μPET scan was performed subsequently. The mice were divided in two groups: an untreated control group (*n* = 6 for LAPC-4 and 22Rv1) and a surgical castration group for AD (*n* = 7 for LAPC-4, *n* = 6 for 22Rv1). Control animals received a sham operation. [^18^F]FDG, [^11^C]choline and [^11^C]acetate μPET imaging was repeated 5 days after surgery (follow-up). The experimental design is illustrated in Figure 1. Preceding μPET scanning, tumour size (mm^3^) was measured using a calliper. Prostate-specific antigen (PSA) plasma levels were determined after baseline but before the start of treatment and after follow-up μPET imaging. At the end of the experiment, the weight of the seminal vesicles and prostate was determined and normalised to the body weight of the animal to control for efficient AD, and histological examination (H&E staining) was performed on isolated tumour tissues.

### μPET/μCT acquisition

**Figure 2:**
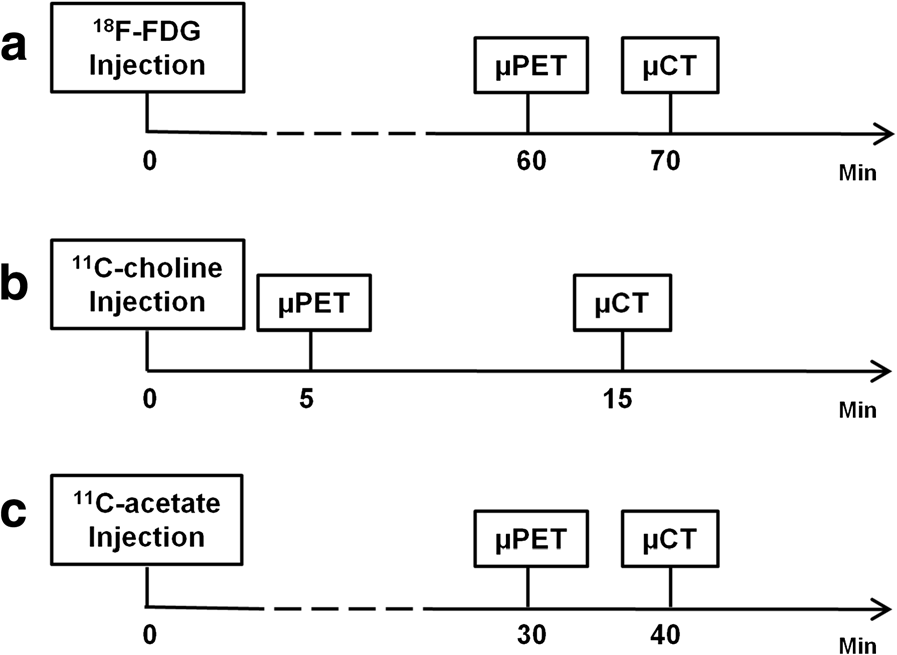
**Schematic illustration of the μPET imaging protocol of (a) [**^**18**^**F]FDG, (b) [**^**11**^**C]choline and (c) [**^**11**^**C]acetate.**

Approximately 4 to 6 weeks after subcutaneous cell inoculation, baseline scanning of the animals was done (Focus 220 microPET, Concorde-CTI/Siemens, Knoxville, TN, USA). Mice were first anaesthetised with 1% to 2% isoflurane, and body weight was determined. Tracer injection was then done via the tail vein before mice were fixed in a designed holder that is compatible for the μPET and μCT scanner and aids the co-registration of both images. The average dose (mean ± SD) of [^18^F]FDG, [^11^C]choline and [^11^C]acetate at the start of μPET imaging was 8.28 ± 0.60, 9.19 ± 1.28 and 4.33 ± 0.53 MBq, respectively. The holder was placed, and tumours were positioned in the field of view of the μPET scanner. Further, a transmission scan was acquired to correct for attenuation. A 10-min static μPET scan of [^18^F]FDG was obtained 1 h post injection for all animals. In advance, mice were fasted for at least 6 h and received an intramuscular injection of 1 mg furosemide (Lasix, Sanofi-Aventis, Diegem, Belgium) at the same time as the tracer injection in order to reduce reconstruction artefacts. μPET imaging was performed using the optimal acquisition times for [^11^C]choline and [^11^C]acetate available from the literature. The optimal scanning interval was determined as the point where, in a dynamic PET acquisition, a steady state was reached. The first two animals of each xenograft model were evaluated, and literature data were confirmed [10,18]. Uptake of [^11^C]choline in the LAPC-4 and 22Rv1 tumour model was determined for 10 min starting 5 min after injection of the tracer. [^11^C]acetate tumour uptake was determined in these animal models during a 10-min static scan 30 min post injection (Figure 2).

Directly after μPET imaging, animals were positioned in the μCT scanner while still fixed to the designed holder. A small-animal CT scanner (SkyScan 1076, Skyscan, Kontich, Belgium) for three-dimensional (3D) tumour localisation and delineation was used. During μCT scanning, the detector and X-ray source (X-ray energy 50 kV) rotated around a fixed bed in a step and shoot mode which allowed the animal to be kept in the same horizontal position as in the μPET scanning device.

### Imaging fusion and quantitative analysis

List mode data of μPET images were converted into 3D sinograms, followed by 3D filtered back projection (FBP). μCT images were reconstructed using a standard protocol and software provided by the manufacturer. The μPET and μCT data sets were imported into PMOD and co-registered using fiducial markers placed on the designed animal holder. Volumes of interest (VOIs) were drawn around the tumour to obtain the maximal standardised uptake value (SUV_max_). In addition to SUV_max_, the mean standardised uptake value with threshold (SUV_meanTH_ with TH = Percentage of SUV_max_) was calculated in all tumours in order to confirm the androgenic effect on tracer uptake. The threshold was set at 65% for [^18^F]FDG and 20% for [^11^C]choline and [^11^C]acetate. Threshold values were chosen empirically in order to approximate a VOI including only viable tumour tissue. To eliminate the dependency on the injected dose and animal weight, SUV values were calculated using the following formula: SUV = Measured radioactivity concentration in the tumour (Bq/g) × Body weight (g)/Injected dose (Bq). The changed SUV in each tumour was calculated as the percentage of the baseline scan on day 0: Percentage change = (SUV_(*x*)_ − SUV_(0)_/SUV_(0)_ × 100%), where SUV_(*x*)_ is the tracer uptake on day *x*.

### Statistical analysis

Statistical analysis was performed using Graphpad Prism (San Diego, CA, USA). For each experimental group, data are expressed as the mean percentage changed SUV ± standard error of the mean (SEM). To evaluate the effect of AD on the uptake of the tracers in androgen-sensitive and androgen-independent PCa, data were analysed with one-way ANOVA and Bonferroni post-hoc testing. For evaluating the effect of AD on prostate and seminal vesicle weight and PSA secretion, mean values were compared using the unpaired Student’s *t* test; *p* values ≤ 0.05 indicate significant differences between the mean values of abovementioned parameters in the control and castration group.

## Results and discussion

### Results

#### Effect of AD on metabolic PET imaging in hormone-naive PCa

**Figure 3:**
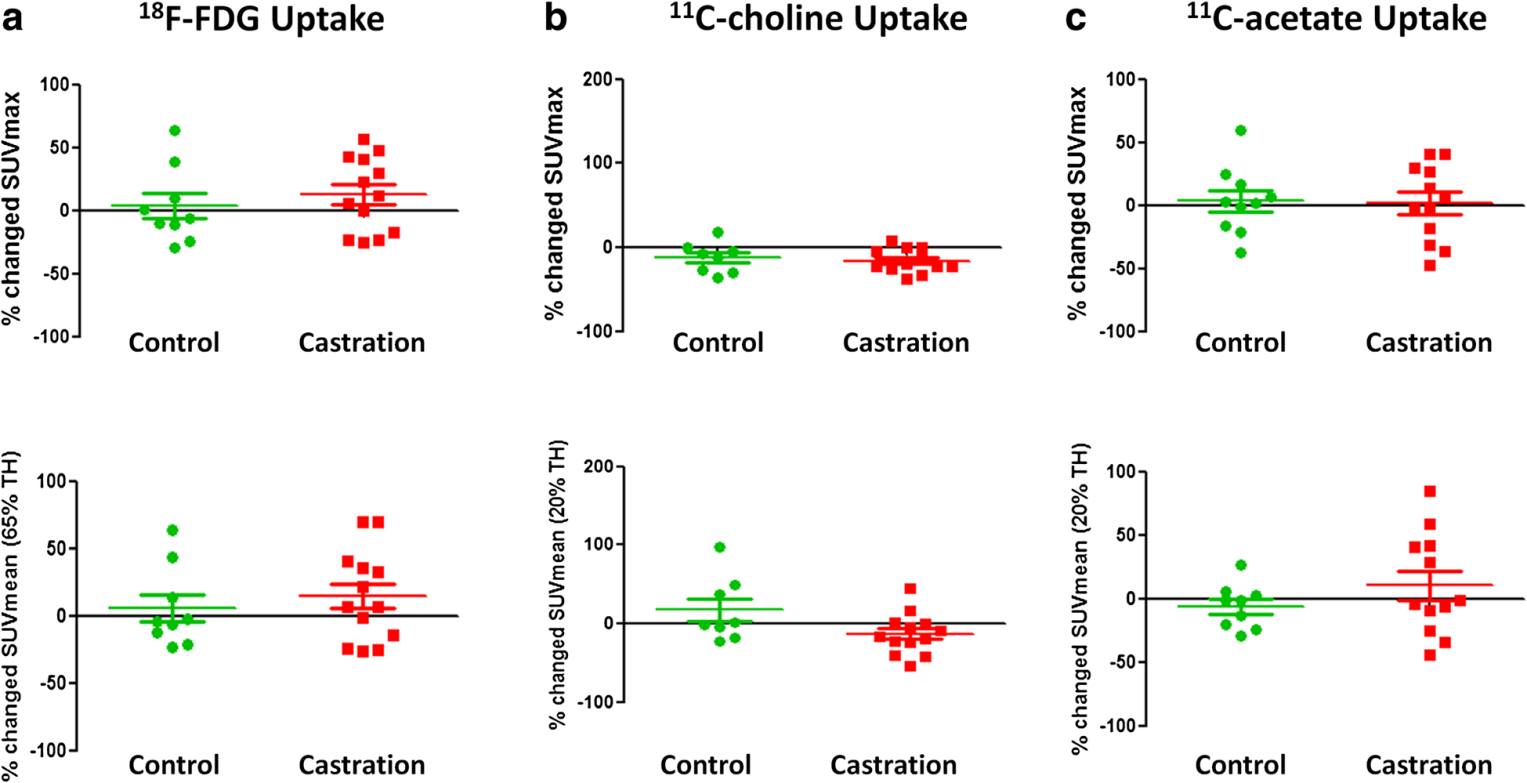
**Percentage changed SUV**_**max **_**and SUV**_**meanTH **_**values of tracer uptake in control and castrated LAPC-4 tumour-xenografted mice.** Percentage changed SUV values of (**a**) [^18^F]FDG, (**b**) [^11^C]choline and (**c**) [^11^C]acetate in control (*n* = 6) and castrated (*n* = 7) LAPC-4 tumour xenografted mice. Data are presented as mean ± SEM. Significant differences between both groups are indicated with *p* < 0.05.

Castration-induced AD did not have a significant effect on metabolic PET imaging as measured by SUV_max_ in hormone-naive LAPC-4 xenografts (Figure 3). Looking into detail, the following results were obtained: in control animals, [^18^F]FDG uptake remained stable over time (3.53 ± 10.12%), whereas a non-significant increased uptake was observed in tumours of castrated animals (12.88 ± 8.20%). As for [^11^C]choline, decreased SUV_max_ values were observed in control and AD-treated animals over time (−12.28 ± 6.25% versus −15.87 ± 3.81%). Similar as for both other tracers, AD did not significantly affect the uptake of [^11^C]acetate in control and treated animals. Moreover, [^11^C]acetate uptake remained stable in both experimental groups (3.57 ± 8.48% versus 1.54 ± 8.73%). Identical results regarding the effect of AD on tracer uptake were obtained with the SUV_meanTH_ values (Figure 3). AD did not significantly decrease the *in vivo* uptake of [^18^F]FDG (5.51 ± 9.92% versus 14.74 ± 9.33%), [^11^C]choline (17.38 ± 14.43% versus −12.95 ± 7.26%) and [^11^C]acetate (−5.98 ± 5.88% versus 10.59 ± 11.48%). However, compared to the uptake in control tumours, [^11^C]choline uptake tended to decrease following castration.

#### Effect of AD on metabolic PET imaging in CRPC

**Figure 4:**
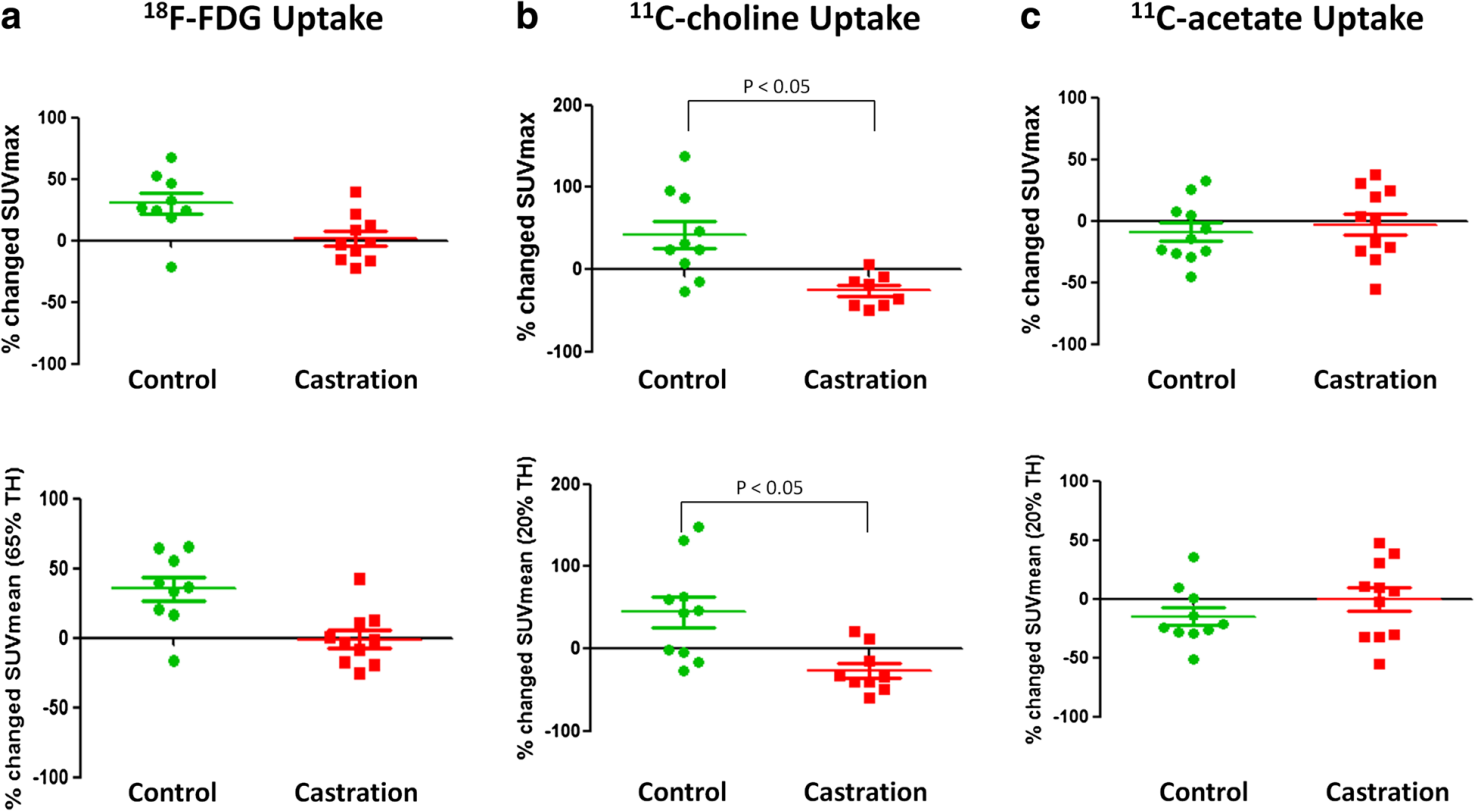
**Percentage changed SUV**_**max **_**and SUV**_**meanTH **_**values of tracer uptake in control and castrated 22Rv1 tumour-xenografted mice.** Percentage changed SUV values of (**a**) [^18^F]FDG, (**b**) [^11^C]choline and (**c**) [^11^C]acetate in control (*n* = 6) and castrated (*n* = 6) 22Rv1 tumour-xenografted mice. Data are presented as mean ± SEM. Significant differences between both groups are indicated with *p* < 0.05.

**Figure 5:**
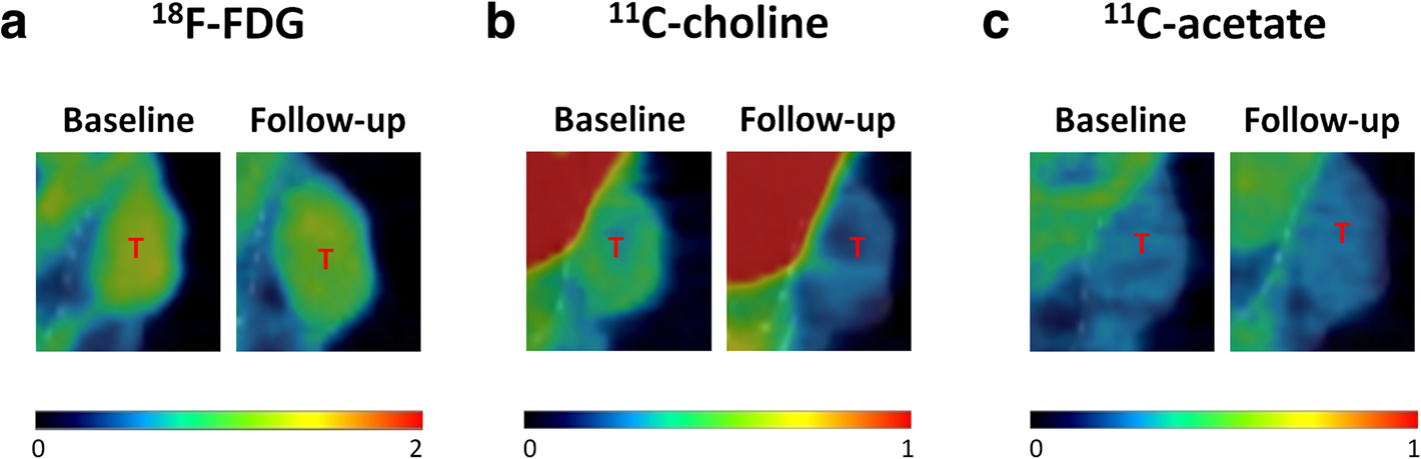
**μPET/μCT image of** [^**18**^**F]FDG (a), [**^**11**^**C]choline (b) and [**^**11**^**C]acetate (c) uptake in a representative 22Rv1 tumour.** SUV_max_ and SUV_meanTH_ were determined in the tumour before and after AD. They demonstrate the inhibition of tracer uptake (stabilised [^18^F]FDG uptake and decreased [^11^C]choline uptake under AD versus increased uptake in control). AD did not affect the uptake of [^11^C]acetate. μPET [^18^F]FDG data are scaled from 0 to 2 SUV, those of [^11^C]choline and [^11^C]acetate from 0 to 1 SUV. T, tumour.

AD decreased the uptake of [^18^F]FDG and [^11^C]choline in the androgen-independent yet responsive 22Rv1 xenograft model (Figure 4a,b). [^18^F]FDG uptake increased over time in the control group (30.20 ± 8.33%), whereas trapping of this tracer remained unchanged upon AD treatment *in vivo* (1.63 ± 6.15%). Like [^18^F]FDG, [^11^C]choline uptake increased over time in control tumours (41.51 ± 16.39%). However, AD significantly reduced the uptake of [^11^C]choline in animals following this treatment (−26.07 ± 6.97%) (Figures 4b and 5). In contrast with both these tracers, [^11^C]acetate uptake in control tumours remained stable (−8.92 ± 7.30%) and did not differ from the uptake in AD-treated animals (−2.97 ± 8.85%) (Figure 4c). Results of the SUV_meanTH_ values are in agreement with those of SUV_max_ (Figure 4). An inhibiting effect of AD was observed on the uptake of [^18^F]FDG and [^11^C]choline (35.32 ± 8.72% versus −0.88 ± 6.27% and 44.30 ± 19.01% versus −26.62 ± 9.06%, respectively), while the *in vivo* uptake of [^11^C]acetate remained unaffected (−14.85 ± 7.71% versus −0.68 ± 9.97%).

#### Tumour growth and histology

**Figure 6:**
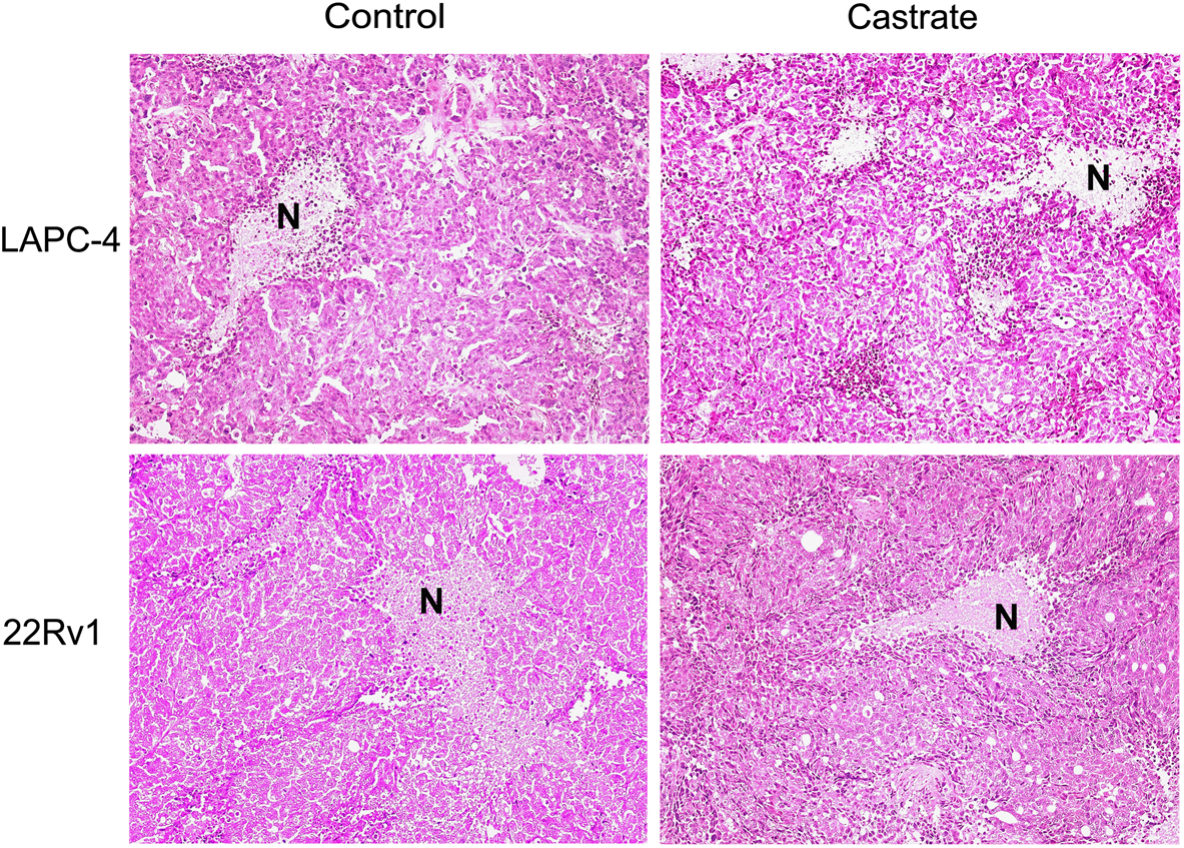
**H&E staining of representative LAPC-4 and 22Rv1 tumour tissues grown in control and castrated animals.** Original magnification is ×100. AD did not affect tumour cell morphology, and necrosis occurred equally in both experimental groups of the animal models. N, necrosis.

Based on calliper measurement, surgical castration did not significantly change the tumour size of LAPC-4 (*p* = 0.1941) and 22Rv1 (*p* = 0.6881) xenografted animals over time. Morphological differences were not observed in viable tumour cells of control compared to those of castrated animals (Figure 6). Also, necrosis occurred equally in control and AD-treated tumours of each PCa model. Inflammation was observed in all tumours, yet this was not increased by AD. In viable tumour tissues, inflammation was absent or barely present, whereas inflammatory cells - polynuclear and, to a lesser extent, mononuclear cells - were observed surrounding or within necrotic areas.

#### PSA secretion and prostate/seminal vesicle weight

Over time, AD significantly diminished PSA secretion of LAPC-4, but not 22Rv1 tumour cells, which indicates the androgen sensitivity of exclusively the LAPC-4 cells *in vivo* (Table 1). Weights of the prostate and seminal vesicles were reduced in treated animals compared to those of control animals (Tables 2 and 3) which demonstrate a successful AD therapy *in vivo*.

**Table 1:** **Average change of PSA secretion (ng/l/mm**^**3 **^**tumour)**

**Cell line**	**Control**	**Castrate**	***p *value**
LAPC-4	−5.05 ± 6.19	−44.29 ± 7.71	0.0026
22Rv1	−0.58 ± 2.29	−0.88 ± 1.75	0.9185

Control versus androgen-deprived hormone-naive LAPC-4 and androgen-independent 22Rv1 xenograft tumours.

**Table 2:** Prostate and seminal vesicle weight 7 days post castration of control and androgen-deprived LAPC-4 xenograft tumours

	**Control**	**Castrate**	***p *value**
Prostate	1.90 ± 0.20	1.47 ± 0.12	0.0780
Seminal vesicles	4.08 ± 0.58	1.69 ± 0.27	0.0023

Prostate and seminal vesicle weight (mg/g body weight).

**Table 3:** Prostate and seminal vesicle weight 7 days post castration of control and androgen-deprived 22Rv1 xenograft tumours

	**Control**	**Castrate**	***p *value**
Prostate	1.68 ± 0.15	1.20 ± 0.07	0.0137
Seminal vesicles	2.91 ± 0.25	1.16 ± 0.04	<0.0001

Prostate and seminal vesicle weight (mg/g body weight).

### Discussion

The current study investigated the short-term impact of castration-induced AD on the uptake of metabolic PET tracers - [^18^F]FDG, [^11^C]choline and [^11^C]acetate - in PCa cells *in vivo*. The percentage changed uptake, based on SUV_max_ and SUV_meanTH_, of each tracer was evaluated in control and AD-treated androgen-sensitive (LAPC-4) and androgen-independent (22Rv1) PCa xenografts in order to assess the impact of androgen on tracer uptake. In the clinical setting, SUV_max_ is usually the preferred value for evaluating therapy response after AD. Nevertheless, this approach quantifies only the highest tumour uptake localised in one voxel, which is not a true characterization of the metabolic status of the entire tumour. For this reason, we additionally looked at the effect of AD on the average tracer uptake in viable tumour cells. A threshold (SUV_meanTH_) was set to exclude uptake values in necrotic tumour tissue, which was equally present in tumours of control and castrated animals as confirmed by histology. Moreover, since tumour sizes were relatively large and the main objective was to accurately quantify tumour uptake, FBP had minor drawbacks as compared to iterative reconstruction while avoiding possible positive bias due to iterative reconstruction [19]. Therefore, all data were reconstructed with FBP using the same reconstruction protocol.

Regarding the image acquisition of [^11^C]choline and [^11^C]acetate, optimal time points following the injection of both tracers were chosen, taking into account background detection and tumoural uptake at a steady state. In the clinical setting, [^11^C]choline PET imaging is preferably acquired 5 min post injection. On the other hand, centres using [^11^C]acetate acquire images 30 min post injection. A high tumour uptake was observed very early after the injection of [^11^C]acetate. However, at that time, tracer uptake was also high in the liver and kidneys. Since background uptake of [^11^C]acetate diminished over time while tumour uptake remained rather stable, a static scan 30 min after tracer injection was the most optimal acquisition time point for tumour imaging with [^11^C]acetate.

Previously, the late-term effect of AD on the uptake of [^18^F]FDG and [^11^C]choline has been characterised in hormone-dependent and hormone-independent PCa xenograft models [20]. The authors demonstrated the lower efficacy of [^11^C]choline PET compared to that of [^18^F]FDG PET for tumour detection and therapy response assessment *in vivo* after surgical castration. Likewise, in this study, not only the trapping of [^11^C]choline, but also of [^11^C]acetate in the xenografted tumours was lower than that of [^18^F]FDG; however, the clinical relevance of [^11^C]choline and [^11^C]acetate and their superiority to [^18^F]FDG for PCa detection have been shown. In the current study, we focussed on evaluating the effect of intratumoural response to AD on PET imaging with both these tracers as well as with [^18^F]FDG [5]. Moreover, in order to minimise partial volume effects in imaging assessment, the xenografted tumours had to have a minimal volume of 150 mm^3^. However, at this point, the proliferation rate of 22Rv1 tumours was rather fast, wherefore long observation periods after AD were not possible. Kukuk et al. evaluated the effect of AD on tracer uptake 2 and 3 weeks following castration [20], which would reflect follow-up imaging of treated PCa in a clinical setting more precisely. However, Oyama et al. demonstrated at an early stage androgen-regulated effects on metabolic PET imaging in androgen-sensitive PCa [18]. In this study, PCa xenografted tumours were androgen-deprived for 1 week by means of diethylstilbestrol (DES) administration. AD significantly diminished the uptake of [^18^F]FDG, but not [^11^C]acetate in androgen-sensitive PCa [18]. Taken these findings into account and the fact that androgen-induced effects on gene expression were previously observed in preclinical models after several days [21,22], we investigated the short-term effect of AD, i.e. 5 days following the start of the treatment, on metabolic PET imaging of PCa xenografted tumours.

In the current study, AD was successful in all animals, but still, no significant change on tumour growth was observed in castrated animals. This observation can however be explained by the fact that AD alters gene expression - biological/molecular changes - rather fast in time, whereas its impact on tumour growth - morphological changes - is mostly a long-term effect. Since all metabolic tracers involved in the study are not specific, the effect of gene alteration on the uptake of the molecular probes might occur later in time. Therefore, an AD-induced inhibition of tumour proliferation, which could have been expected in especially the androgen-sensitive LAPC-4 model, was probably not detected in the present study. Also, we evaluated androgen-mediated effects by means of castration on the uptake mechanisms of metabolic tracers. Although surgical castration significantly reduces testicular androgen synthesis, adrenal androgen, which provides 10% of the total androgen production, remains present. Additionally, in tumour cells, bioconversion of the adrenal androgen androstanediol to dihydrotestosterone takes place of which the latter compound causes AR transactivation [23]. Hence, surgical castration is not equivalent to medical treatment, i.e. AR antagonists, which reduces androgen activity locally and in turn also limits the effect of adrenal and tumoural androgen. Therefore, medical treatment could further extend the effect of AD on tumour proliferation and PET imaging.

Concerning the effect of androgen on PET imaging of PCa patients, AD using bicalutamide has been shown to significantly decrease the uptake of [^11^C]choline in patients undergoing PET/CT for preoperative staging of PCa [12]. Unlike that in patients, [^11^C]choline uptake was not significantly reduced in AD-treated compared to untreated hormone-naive xenografts (LAPC-4) in this experiment. Because LAPC-4 cells are characterised by the expression of the wild-type AR, they are assumed to have a homeostatic AR signalling. A significant androgen-controlled effect on choline metabolism has further been observed in androgen-sensitive, wild-type AR-expressing PCa cell lines [24,25]. Based on these *in vitro* observations, AD-induced alterations on metabolic imaging could have been expected in the current study. Moreover, Jadvar et al. evaluated AD-induced effects on [^11^C]choline uptake in androgen-sensitive PCa xenografted tumours using autoradiography [26]. Tumour uptake of [^11^C]choline was determined 5, 10 and 20 min post injection. Results demonstrated a significantly higher tracer uptake in tumours of castrated animals at only the first two time points. Compared to our study, AD treatment was induced before androgen-sensitive tumour cells were injected subcutaneously and thus before solid tumours were developed. Although differences in [^11^C]choline uptake in these tumours were assumed to result from androgen action, it is not obvious whether this is completely true. In fact, androgen-sensitive cells injected into an androgen-depleted environment are exposed to additional stress as compared to those cells injected into an androgen-rich environment (control animal). Since PSA expression was not monitored in this study and no baseline [^11^C]choline uptake in the cells could be measured, it could be questioned whether androgen-sensitive cells grown under AD developed a truly androgen-sensitive solid tumour after certain weeks. Hence, this would hamper the interpretation of AD-induced effects on [^11^C]choline uptake in androgen-sensitive PCa. Also, autoradiography quantifies the tracer uptake in several tissue slices of a certain organ, while PET offers a 3D image of a region of interest wherefore tracer internalisation in a specific tissue compartment of a living subject can be approached more precisely. The experimental set-up and imaging method used differed from those chosen in our current study, which is most likely the reason for different results obtained.

In patients with recurrent PCa, the development of biochemical failure during AD is observed over time. This stage of the disease - CRPC - is characterised by an increasingly androgen-independent proliferation [27]. Nevertheless, androgen may still affect the expression of genes controlled by AR signalling and the progressive behaviour of tumours. Therefore, at this stage of the disease AD could diminish the uptake of metabolic PET tracers which in turn would suggest a favourable therapy response and delay the detection of recurrences. Hence, this study also examined androgen-induced effects on metabolic PET imaging of the androgen-independent yet androgen-responsive PCa xenograft model 22Rv1. AD decreased the uptake of [^18^F]FDG and significantly of [^11^C]choline *in vivo*, while tumour histology in control and castrated animals was similar; however, [^11^C]acetate PET imaging remained unaffected in both experimental groups. These observations are in line with our previous findings demonstrating an androgen-induced increased uptake of [^18^F]FDG and [^11^C]choline in 22Rv1 cells [25]. Although [^11^C]choline PET is currently used for the detection of recurrent PCa before and following AD treatment of PCa, it is not yet clear whether AD should be withdrawn on a regular basis before [^11^C]choline PET is performed in androgen-resistant PCa. A recent study of Fuccio et al. demonstrated a diminishing effect of AD on the uptake of [^11^C]choline in recurrent PCa which supports the retraction of AD therapy before follow-up imaging in order to increase the sensitivity of [^11^C]choline PET [13]. The current results support these findings, and it is noteworthy that caution may need to be taken for imaging recurrent PCa undergoing biochemical failure during or following AD.

AD treatment by means of surgical castration or AR antagonist both affect tumour viability, which can be detected by nuclear imaging techniques. However, in order to compare tracer internalisation in AD-treated versus non-treated animals accurately, tracer metabolism should remain as far as possible unaffected by AD. The biodistribution of [^18^F]FDG and [^11^C]acetate was studied in control and DES-treated animals [18]. In healthy rats and tumour-bearing mice, AD did not alter tracer clearance from the blood. Also, the uptake of both tracers in background tissue compartments such as the liver, muscle and heart was not significantly affected [18]. Therefore, the biodistribution profile of [^18^F]FDG and [^11^C]acetate is assumed to remain unchanged after AD. This would mean that tracer clearance should not affect PET imaging. Regarding [^18^F]FDG PET, these data were further confirmed by Jadvar et al. [28]. In a later study of this group, [^11^C]choline biodistribution was studied in castrated and non-treated tumour-bearing mice using autoradiography. The authors concluded that AD did not change clearance of [^11^C]choline [26]. In our present study, we did not observe an altered clearance of [^18^F]FDG, [^11^C]choline and [^11^C]acetate following castration, which supports these previously obtained results.

Besides therapy, micro-environmental factors play an important role in tumour behaviour and can affect the evaluation of metabolic PET imaging following the treatment of PCa. Necrosis and inflammation, of which the latter is a main therapeutic effect, interfere with therapy response assessment using PET. However, in this study, both micro-environmental factors were equally observed in tumour tissues of control and castrated animals. Taking this into account and also the observation that AD did not affect [^11^C]acetate uptake in androgen-sensitive and androgen-independent xenografted tumours, this tracer may be applicable for assessing tumour viability after AD treatment at all stages of PCa. The potential use of [^11^C]acetate PET as well as its advantage over the currently mostly employed [^11^C]choline PET for the evaluation of advanced PCa following AD should be considered. However, because the uptake of [^11^C]acetate was rather low and did not visualise proliferation as well as that of [^18^F]FDG and [^11^C]choline, we denote the significance of further preclinical and clinical evaluation in order to confirm the diagnostic efficacy of [^11^C]acetate PET for detecting the viability of PCa lesions before and after therapy.

## Conclusions

AD reduced the uptake of [^18^F]FDG and [^11^C]choline in treated versus untreated androgen-independent yet androgen-responsive PCa. Androgen did not affect metabolic PET imaging of androgen-sensitive tumours. Therefore, [^18^F]FDG-PET and especially [^11^C]choline-PET, which is preferred for the detection of recurrent PCa, could underestimate the presence of viable lesions in patients undergoing biochemical failure early after AD therapy. Since [^11^C]acetate uptake seemed to be independent of androgen *in vivo*, this radiopharmaceutical may be advantageous for detecting PCa following AD. However, it is mandatory to further investigate the clinical significance of metabolic PET imaging with [^11^C]acetate in the context of detecting tumour viability following AD.

## Competing interests

The authors declare that they have no conflict of interest.

## Authors’ contributions

KME designed the experimental protocol, carried out the cell culture and development of the xenograft models, participated in and monitored the imaging of all animals and drafted the manuscript. JVS participated in the study design and helped monitoring the development of cell cultures and xenograft models. EL coordinated and interpreted the histological examination of all xenograft tissue specimens. MK, LM and FMM participated in the design of the study and data analysis. All authors contributed to the draft of the manuscript. All authors read and approved the final manuscript.
